# Ultrasensitive Terahertz Label-Free Metasensors Enabled by Quasi-Bound States in the Continuum

**DOI:** 10.34133/research.0483

**Published:** 2024-09-26

**Authors:** Ride Wang, Lingyu Song, Hao Ruan, Quanlong Yang, Xiao Yang, Xiaobao Zhang, Rundong Jiang, Xiangmin Shi, Alexander P. Shkurinov

**Affiliations:** ^1^ Innovation Laboratory of Terahertz Biophysics, National Innovation Institute of Defense Technology, Beijing 100071, China.; ^2^ Navy Clinical College, Anhui Medical University, Beijing 100048, China.; ^3^ The Fifth School of Clinical Medicine, Anhui Medical University, Hefei 230032, China.; ^4^ Department of Cardiology, The Sixth Medical Center of PLA General Hospital, Beijing 100048, China.; ^5^ School of Physics, Central South University, Changsha 410083, China.; ^6^ Department of Physics and International Laser Center, Lomonosov Moscow State University, Leninskie Gory 1, Moscow 19991, Russia.

## Abstract

Advanced sensing devices based on metasurfaces have emerged as a revolutionary platform for innovative label-free biosensors, holding promise for early diagnostics and the detection of low-concentration analytes. Here, we developed a chip-based ultrasensitive terahertz (THz) metasensor, leveraging a quasi-bound state in the continuum (*q-*BIC) to address the challenges associated with intricate operations in trace biochemical detection. The metasensor design features an open-ring resonator metasurface, which supports magnetic dipole *q*-BIC combining functionalized gold nanoparticles (AuNPs) bound with a specific antibody. The substantial enhancement in THz–analyte interactions, facilitated by the potent near-field enhancement enabled by the *q*-BICs, results in a substantial boost in biosensor sensitivity by up to 560 GHz/refractive index units. This methodology allows for the detection of conjugated antibody–AuNPs for cardiac troponin I at concentrations as low as 0.5 pg/ml. These discoveries deliver valuable insight for AuNP-based trace biomolecule sensing and pave the path for the development of chip-scale biosensors with profound light–matter interactions.

## Introduction

Ultrasensitive biosensors capable of detecting ultralow concentration analytes down to trace levels are in great demand in early diagnostics and real-time monitoring of disease progression [1,2]. Traditional biological detection methods face challenges such as being cumbersome and limited by trace high-sensitivity detection [3,4]. Moreover, these methods necessitate labeling, thereby restricting their applicability in further biological analyses. Interestingly, numerous biomolecules possess vibration and rotation modes within the terahertz (THz) frequency range, exhibiting distinctive molecular fingerprint spectra [5,6]. Leveraging this characteristic, various biomolecules can be identified through THz time-domain spectroscopy visualization, offering label-free and nondestructive capabilities. This technology has garnered widespread attention in the biomedical field [7], targeting a wide range of entities from bacteria [8,9], cells [10–12], and proteins [13–15] to nucleic acids [16], with potential applications for the rapid detection of cardiac troponins.

Nevertheless, the mismatch between THz wavelengths and molecular dimensions results in a weak THz–matter interaction, which limits the application of THz spectroscopy in trace detection [17]. Introducing metasurface enhanced by a strong electromagnetic near field provides an avenue to overcome the bottleneck of THz sensing [18–22], enabling the detection of subtle changes in environmental refractive index and quantifying trace molecules. Great efforts have been put into the study of metasurface-enhanced THz sensing, including surface lattice resonances [22,23], plasmonic Fano resonances [24], guided-mode resonances [25], surface waves [15], and electromagnetic-induced transparency [8–11]. However, the ohmic and radiation losses of these metasurfaces contribute to the low *Q*-factor of resonances based on these electromagnetic modes, limiting the application of metasurfaces in detecting low-concentration analytes [18]. Recently, ultrahigh-*Q* resonances known as quasi-bound states in the continuum (*q-*BIC) with giant localization of electromagnetic energy in small volumes have been integrated into the metasurface to enhance the detection capability for even lower abundance samples [19,26,27]. Given the intricate nature of biological samples, detection schemes based on refractive index sensing make it difficult to specifically identify target biomolecules among numerous biological molecules [28]. Therefore, there is a pressing need for advancements in specific biosensing based on *q-*BIC THz metasurfaces.

**Fig. 1.**
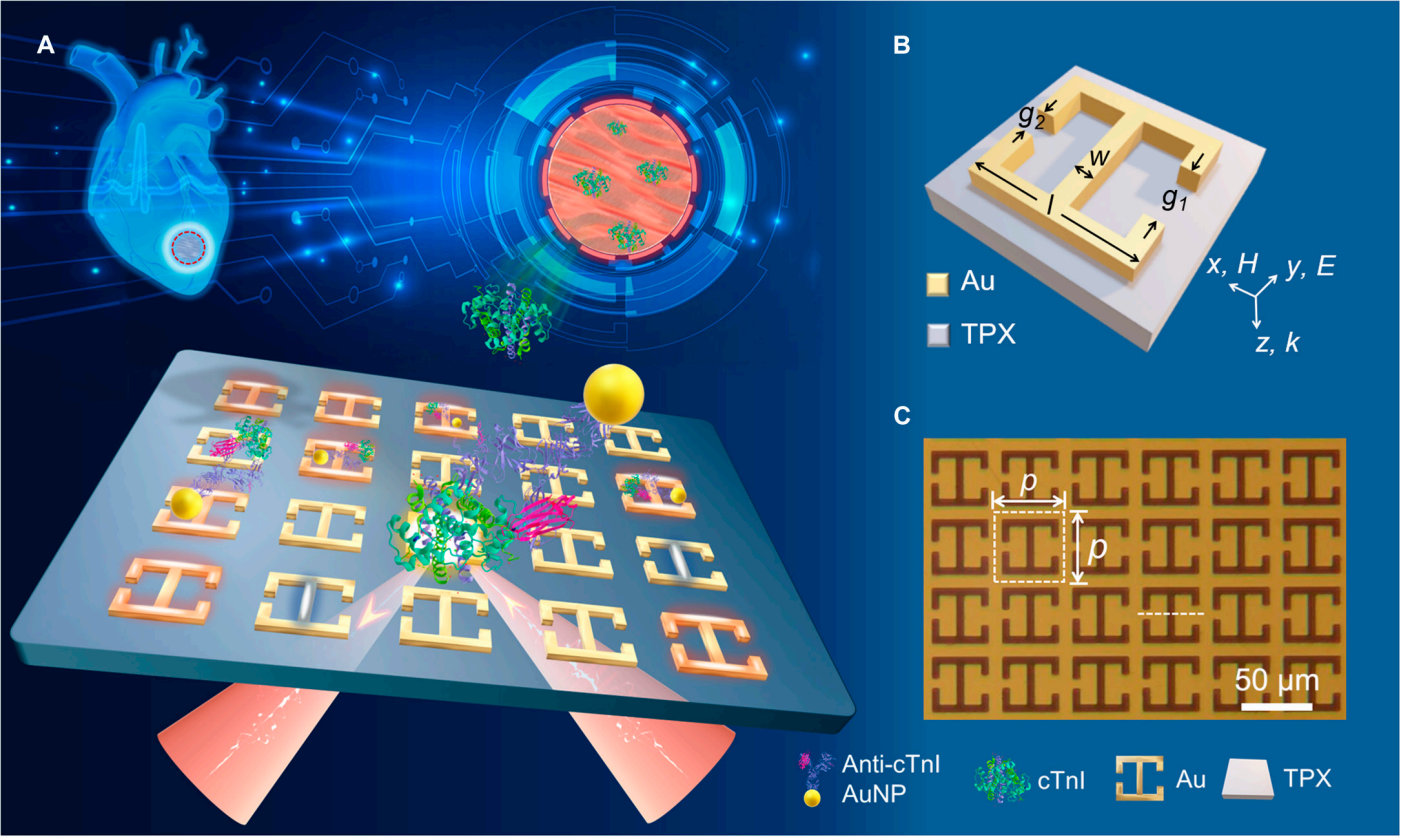
Schematic of the THz plasmonic ultrasensitive biosensor. (A) THz *q*-BIC metasensor for trace troponin detection based on biological functionalization. (B) Geometrical configuration of the gold meta-atom on the polymethyl pentene (TPX) substrate within one unit cell: *l* = 40 μm, *w* = 5 μm, *g*_1_ = 14 μm, and *g*_2_ is variable for asymmetry. (C) Micrograph of the fabricated metasurface sensor at the lower right with the unit cell: *p* = 50 μm.

Herein, we introduce an ultrasensitive THz plasmonic metasensor by harnessing the magnetic dipole (MD) *q*-BIC in conjunction with functional colloidal gold nanoparticles (AuNPs) (Fig. 1). The constructed metasurfaces comprised periodically arranged open-ring resonators, with the asymmetry of the resonant structure allowing for precise tailoring of the linewidth and *Q*-factor of the *q*-BICs. The biologically functionalized AuNPs, decorated with specific antibodies, selectively target the proteins of interest through noncovalent interactions. The *q*-BIC mode exhibits a remarkable enhancement of local electromagnetic energy, amplifying the light–analyte interaction well beyond what is achievable by traditional resonance modes, resulting in a high sensitivity of up to 560 GHz/refractive index units (RIU). Subsequent biological experiments demonstrate the capacity of metasensor to specifically identify trace levels of cardiac troponin down to the pg/ml level. This study establishes a promising foundation for detecting myocardial injury biomarkers and holds the potential for expansion to a diverse array of THz biosensors.

## Results

### Structural characterization of the *q*-BIC metasurface

The proposed metasurface employs a design featuring metal open-ring resonators, with the geometric shape and corresponding parameters of the metasurface atoms (Fig. 1). The gold open-ring resonators are positioned on a polymethyl pentene (TPX) substrate, with a rectangular lattice period of *p* × *p* = 50 μm × 50 μm, and a TPX substrate thickness of 2 mm. In the *x*–*y* plane, the rest size of the gold meta-atoms is as follows: a side length of *l* = 40 μm, a width of *w* = 5 μm, and gaps of *g*_1_ = 14 μm, respectively. In particular, the gap width *g*_2_ serves as a critical parameter for realizing *q*-BIC modes. By breaking the structural symmetry in the *y*-direction, the energy of the bound states leaks into the continuum spectrum, resulting in the transition of nonradiative BIC to observable high-*Q q*-BIC [27]. Moreover, TPX exhibits negligible intrinsic material losses in the THz range, thus mitigating the impact of the substrate on *Q*-factors of *q*-BIC.

**Fig. 2.**
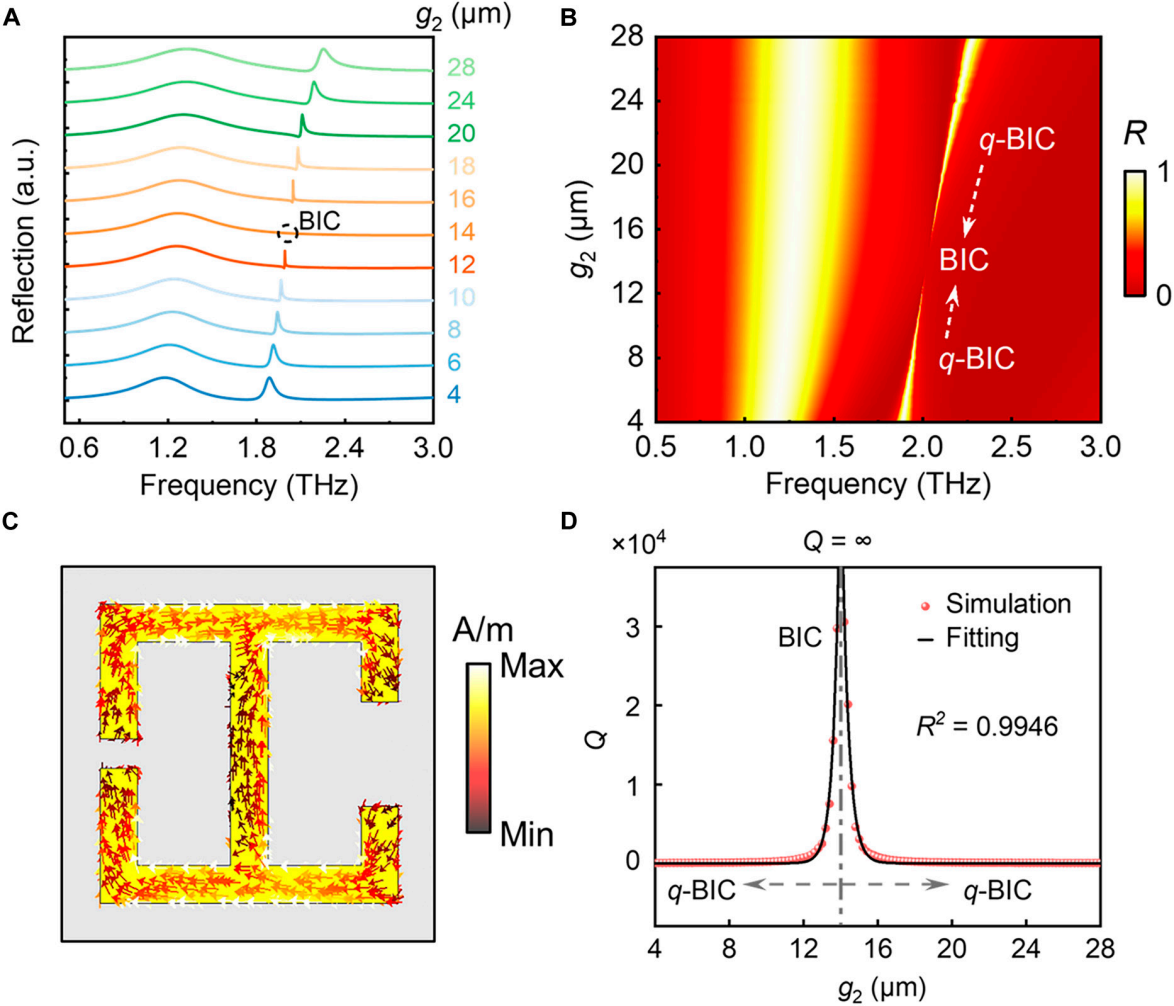
The electromagnetic resonance mode analysis of THz metasurface. (A and B) Simulated reflectance spectra at representative asymmetry degrees with different gap *g*_2_. (C) The simulated current density distribution with *g*_2_ = 4 μm. (D) The nonlinear fitting *Q*-factor of the simulated *q*-BIC resonance as a function of *g*_2_ with the coefficient of determination *R*^2^ > 0.99.

To demonstrate the spectral response and resonance characteristics of the metasurface, numerical calculations are carried out using the frequency domain solver of the CST studio suite. Floquet periodic conditions are set in the *x*- and *y*-directions, while the open boundary condition is taken in the *z*-direction. The electric field of the excitation THz wave is along the *y*-direction to model the polarized plane wave propagating along the direction of the exciting field. The sizes of mesh are set smaller than the minimum construct size in each direction for convergence results with precision. The substrate material is set as a lossless medium and the resonator is modeled as a perfect electric conductor (PEC). The simulated result of the *q*-BIC-based structure with different gap widths *g*_2_ is shown in Fig. 2A and B. When *g*_2_ = *g*_1_ = 14 μm, the resonance higher-order mode is transformed from *q*-BIC to BIC mode. Under these conditions, the theoretical linewidth of the ideal BIC tends to be infinitesimal. The symmetry of the geometric structure is broken by the shift of the opening size *g*_2_ away from 14 μm, the symmetric protected BIC is transformed into Fano line-shape *q*-BIC with a finite *Q*-factor, and the linewidth of the accompanying resonance with a lower frequency remains constant.

The surface charge associated with the *q*-BIC resonance (*g*_2_ = 4 μm) primarily accumulates along the edges of the resonator and within the wider gap region, corresponding to the electromagnetic energy concentrates on the edges of the resonator, implying a heightened potential for interaction with trace analytes (Fig. 2C). The excellent match between the computational analysis and nonlinear fitting illustrates the behavior of the *Q*-factor from perfect BIC to *q*-BIC (Fig. 2D). The *Q*-factor of perfect BIC approaches infinity, while *q*-BIC drops to lower than 10 as the gap size *g*_2_ deviates from 14 μm. Here, the *Q*-factor is defined as *Q* = *f*_R_/FWHM, where *f*_R_ represents the center frequency of the resonance, and FWHM is the full width at half maximum of the resonant peak.

### Sensing mechanism of the *q*-BIC metasurface

**Fig. 3.**
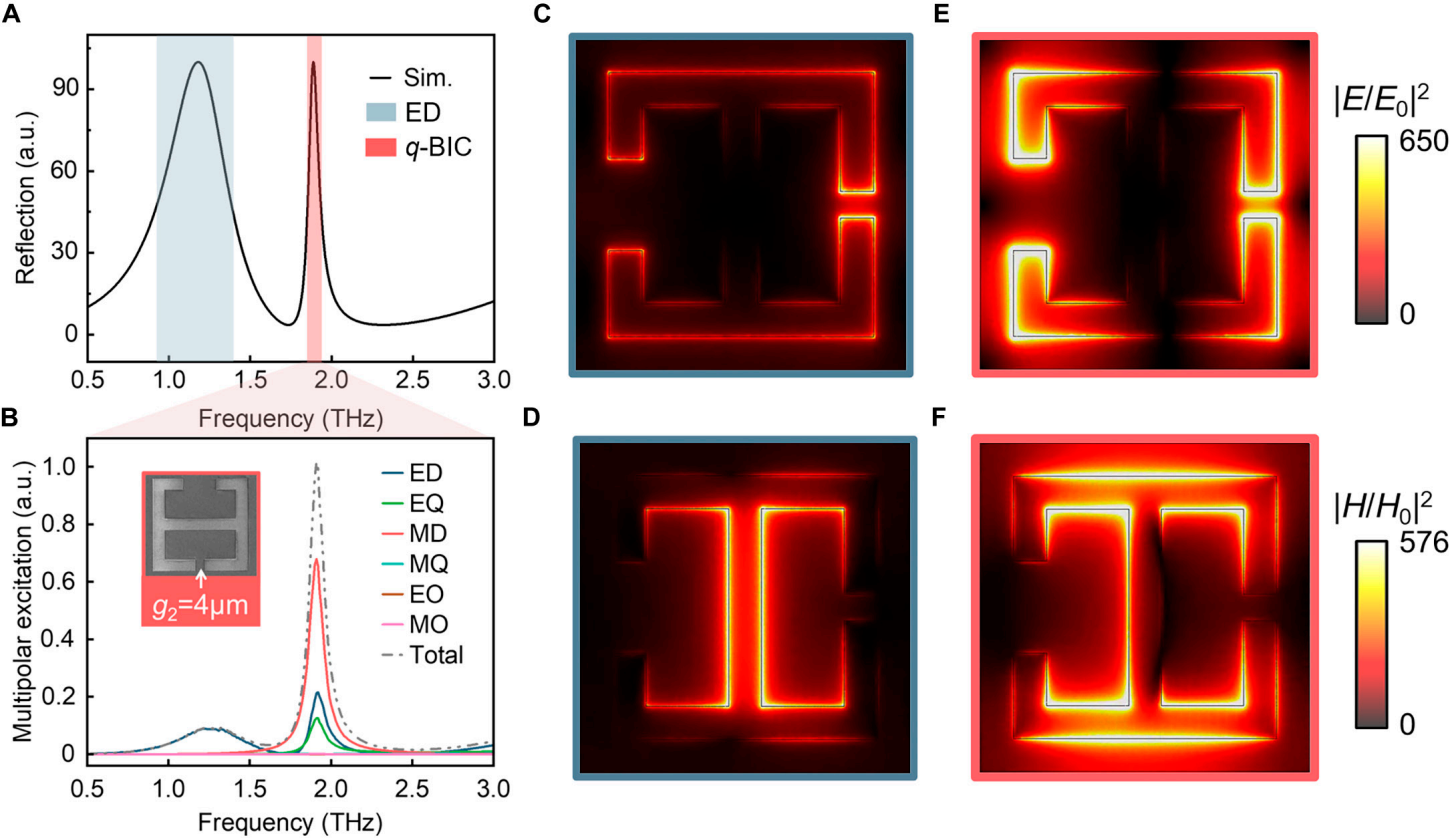
Normalized electromagnetic near-field distribution. (A) Calculated reflection spectrum of the unit cell with a structure parameter *g*_2_ = 4 μm. (B) Spherical multipolar decomposition result when *g*_2_ = 4 μm. The *q*-BIC mode is mainly contributed by the MD, while another resonance is the ED. The inset shows the fabricated samples corresponding to the condition of *g*_2_ = 4 μm. (C and E) Comparison of electric near-field intensity enhancement |*E*/*E*_0_|^2^ between the ED mode (1.18 THz) and *q*-BIC mode (1.89 THz), where |*E*_0_| represents the incident field amplitude. (D and F) Simulated magnetic near-field intensity enhancement |*H*/*H*_0_|^2^ for *g*_2_ = 4 μm, where |*H*_0_| denotes the incident magnetic field amplitude. These results indicate that the *q*-BIC mode has a stronger electromagnetic near field and therefore higher surface sensitivity.

To clarify the contributions of different electromagnetic modes to the 2 resonances, we conducted a multipole decomposition in spherical coordinates for the metasurface [29]. Here, we primarily considered low-order terms of multipole expansion, including electric dipoles (ED), electric quadrupoles (EQ), MD, magnetic quadrupoles (MQ), electric octupoles (EO), and magnetic octupoles (MO). To clearly elucidate the contribution of multipoles, *g*_2_ = 4 μm was chosen for the analysis of electromagnetic modes. For the 2 resonant modes observed at *g*_2_ = 4 μm as depicted in Fig. 3A, it is more distinctly visible in Fig. 3B that the low-order modes are predominantly sustained by the ED mode, whereas the high-order *q*-BIC modes are primarily driven by the MD mode with a minor contribution from ED and EQ modes. The interference of scattering between even and odd modes governs the resonance characteristics of *q*-BIC. As *g*_2_ increases to 8 μm, similar conclusions are drawn from the multipole excitation analysis, yet the varying scattering interference of even and odd modes alters the electromagnetic energy leakage, consequently affecting the *Q*-factor of the *q*-BIC (Fig. S2, Supplementary Materials).

The comparison between the electromagnetic field distributions of the ED mode and the *q*-BIC mode is illustrated in Fig. 3C to F. The electric field and magnetic field distributions of the 2 resonances are simulated and normalized to determine the intensity enhancement |*E*/*E*_0_|^2^ and |*H*/*H*_0_|^2^, where |*E*_0_| and |*H*_0_| represent the incident field amplitude. The near-field intensity enhancement of the *q*-BIC reaches up to 650 and 576, respectively. These observations suggest that the *q*-BIC exhibits a more robust electromagnetic localization, thereby enhancing sensing sensitivity. Furthermore, the charge density distribution of the 2 resonances (Fig. S2, Supplementary Materials) sheds light on the extensive and intense interaction between light and analytes associated with the *q*-BIC resonance.

**Fig. 4.**
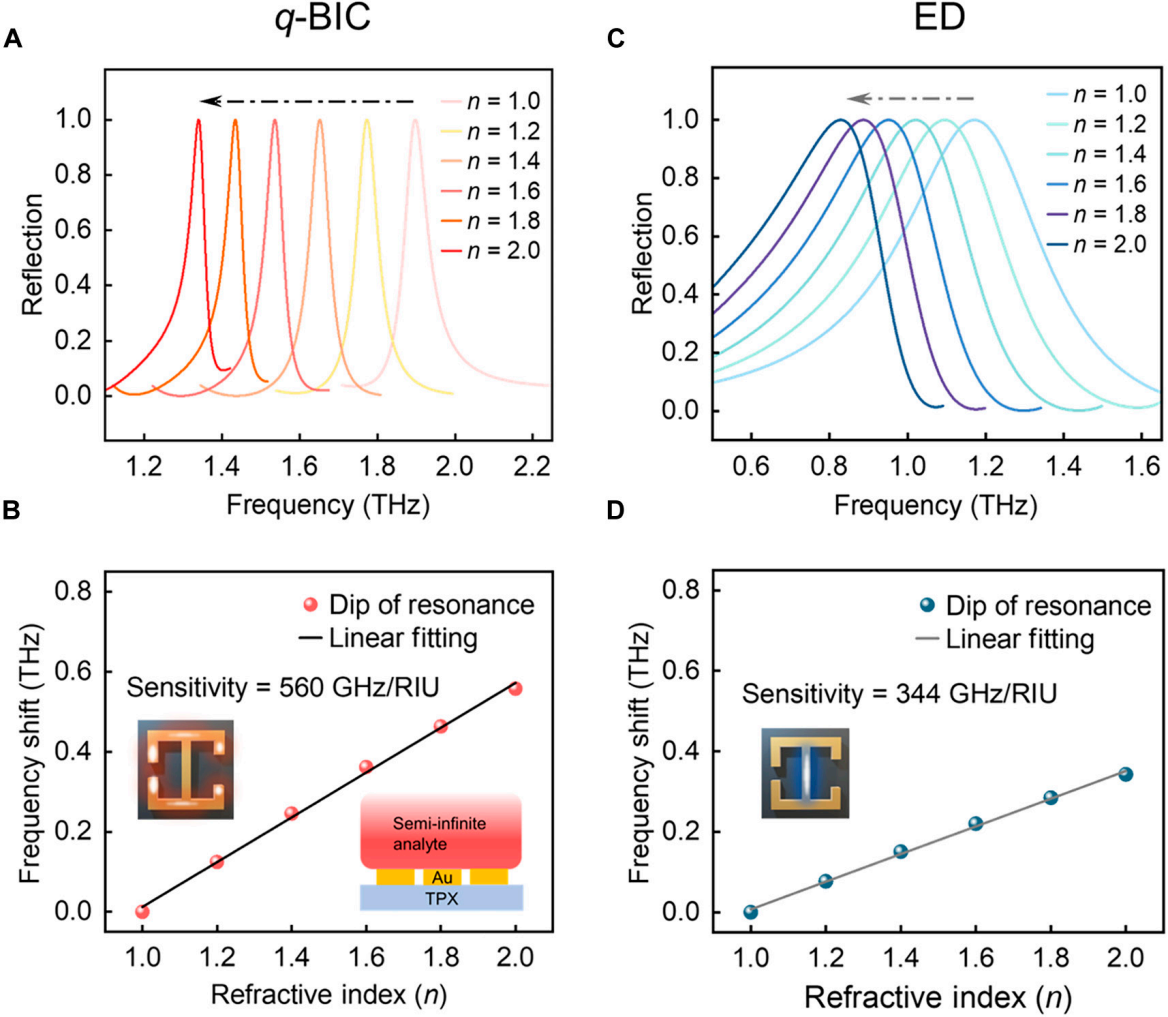
Comparison of sensing performance between the 2 resonant modes. (A and B) Frequency shift and linear fitting of *q*-BIC resonant mode with different refractive index. The sensitivity of *q*-BIC resonance reaches 560 GHz/RIU. (C and D) Reflection spectra and linear fitting of ED resonant mode with the refractive index *n* varies from 1.0 to 2.0. The dotted arrows denote the frequency shift as the refractive index increases.

To study the optical sensing performance of the proposed metasurface, semi-infinite thickness analytes were used for simulation [14]. The refractive index ranges from *n* = 1.0 to *n* = 2.0, covering biomedical materials used in THz sensing research, to compare the sensing performance of the *q*-BIC mode and the ED mode. Figure 4A and C show the frequency shift in reflected spectra of *q*-BIC and ED (*g*_2_ = 4 μm), respectively. The sensitivity of the refractive index biosensor *S* is defined as Δ*f*/Δ*n*, where Δ*f* is the resonant frequency shift when the analyte is placed on the metasurface and Δ*n* represents the refractive index of the simulated analyte. The electromagnetic energy of the *q*-BIC mode is concentrated at the edge of the resonator, whereas that of the ED mode is located at the joint in the middle of the resonator (Fig. 3). It is clear from Fig. 4B that the sensitivity of the *q*-BIC mode reaches 560 GHz/RIU, surpassing the 344 GHz/RIU of the ED mode depicted in Fig. 4D. This further illustrates the superior sensing capability of the *q*-BIC resonance compared to the ED resonance.

**Fig. 5.**
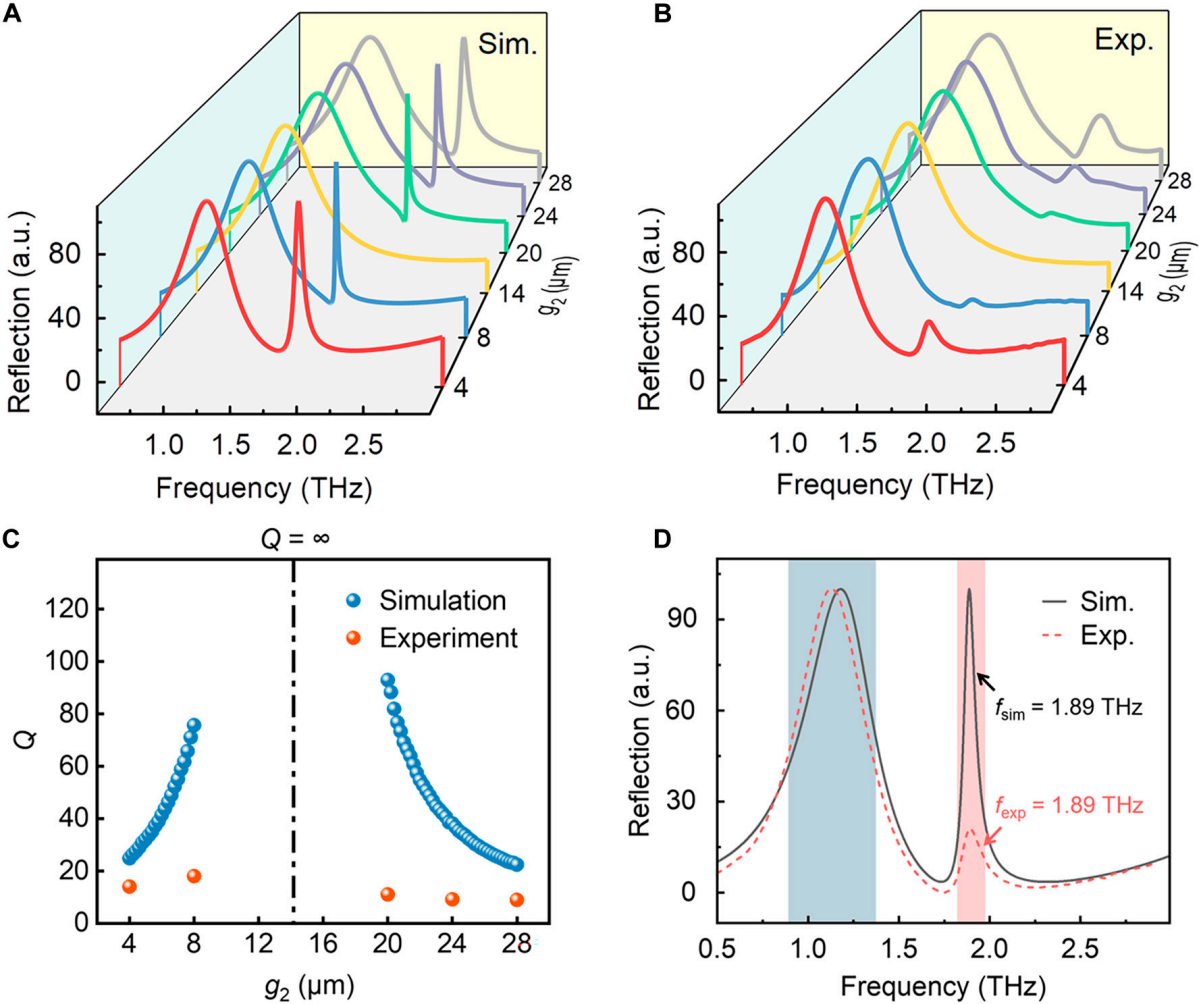
THz sensor evaluation of trace cardiac troponin measurements. (A and B) Simulation calculations and experimental measurements of reflection spectra with *g*_2_ = 4, 8, 14, 20, 24, and 28 μm, respectively. (C) *Q*-factor of *q*-BIC mode from the simulation and the experiment. (D) Comparison of reflection spectra between the simulation and the experiment when *g*_2_ = 4 μm.

To experimentally demonstrate the spectra signature of the proposed metasurface (Fig. S3, Supplementary Materials), we fabricated the metasurfaces using lithography techniques and magnetron sputtering methods, and the optical image of the fabrication sample is shown in Fig. 1C. Here, 6 typical gap widths *g*_2_ = 4, 8, 14, 20, 24, and 28 μm are selected to demonstrate the performance of *q*-BIC in Fig. 5B. The THz time-domain pulse signals are measured from the back of the sensor on the THz time-domain spectroscopy system at dry room temperature. The THz transmission results of the sample (*E*_s_) and the reference object (*E*_r_) are obtained by standard Fourier transform, and then the normalized reflection spectrum |*E*_s_/*E*_r_|^2^ of the sample is obtained by normalization based on the reference signal. Figure 5A and B display the simulated and experimental results of reflection spectra from these 6 samples. The behavior of the *Q*-factor from the experiment results is in agreement with the simulated one except for the smaller value of the *Q*-factor. Moreover, the evolution of experimentally measured *Q*-factors in Fig. 5C still follows the law obtained by simulation, and the highest measured *Q*-factor reaches 110. Interestingly, the measured central frequency of *q*-BIC with *g*_2_ = 4 μm matches well with the simulated frequency, which can be seen from Fig. 5D; thus, we employed this metasurface for subsequent cardiac troponin measurement.

### Trace detection of cardiac troponin I

**Fig. 6.**
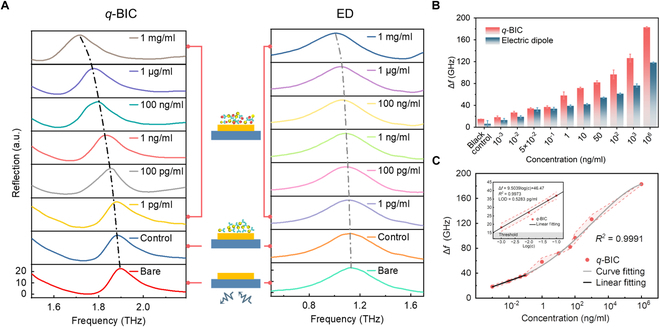
Evaluation of trace cardiac troponin measurements. (A) Reflection spectra of the ED mode (left) and *q*-BIC mode (right) at different concentrations of cardiac troponin I (cTnI) solution. (B) Full display of 2 peak frequency shifts, showing a smaller insert intuitively demonstrating the relative position of the *q*-BIC mode and ED mode. (C) Fitting curve of the *q*-BIC frequency shift.

Next, in order to exploit the potential of *q*-BIC for intense light–matter interaction, we have implemented the ultrasensitive detection of trace cardiac troponin I based on the proposed *q*-BIC metasurface. Cardiac injury constitutes a significant contributing factor to global mortality. Rapid detection of cardiac-specific troponins is vital for precise assessment of myocardial damage, particularly in cases of myocardial infarction and acute heart failure. The middle column in Fig. 6A illustrates the process of specimen handling and experimental testing. Recombinant antibodies are selected for immune preparation attributed to their elevated affinity, high sensitivity, and minimal cross-reactivity in binding to antigens [30]. It is imperative to note that by utilizing high-order BICs, the detection limit could potentially be extended to 1 fM [31]. AuNPs possess a substantial near-field enhancement; hence, immunocolloidal gold is introduced for detection. A sufficiently reactive solution with varying concentration gradients was prepared for detection [22]. The unloaded metasurface serves as the control group, while immunocolloidal AuNPs with zero concentration are utilized as the control group.

We provide intuitive demonstrations of the resonance redshifts for the ED mode (Fig. 6A, left) and the *q*-BIC mode (Fig. 6A, right) at diverse concentrations of the evaporated specimen. Figure 6B displays the frequency shifts of both modes, underscoring the superior sensing capability of the *q*-BIC mode. To further elucidate these findings, a fitting analysis of the frequency shift of the *q*-BIC mode is conducted, as shown in Fig. 6C. Notably, there exists a robust linear correlation between the frequency shift and the logarithm of cTnI concentrations (log(c)) within the range of 1 to 100 pg/ml, yielding the linear regression equation Δ*f* = 9.5039 log(c) + 46.47 (*R*^2^ = 0.9973). Factoring in the blank control signal mean and triple standard deviation, the limit of detection (LOD) is determined to be 0.5 pg/ml. When compared with the biosensors reported for troponin detection in Table, our proposed metasurface exemplifies outstanding sensing performance for low-concentration analytes, achieving detection limits that are an order of magnitude lower than existing methods.

**Table.** The list of performance parameters of troponin biosensors

Detection approach	Target	LOD	Reference
Chemiluminescence method	S_100_A_1_	130 pg/ml	[29]
Fluorescence analysis	cTnI	16 pg/ml	[30]
Fluorescence analysis	Myoglobin	7,600 pg/ml	[3]
Acoustic wave-based approaches	cTnI	6.7 pg/ml	[34]
Electrochemical assays	cTnI	5 pg/ml	[35]
Electrochemical assays	cTnT	8 pg/ml	[36]
Electrochemical assays	cTnT	5,000 pg/ml	[37]
New spectrum analysis	cTnT	500 pg/ml	[38]
New spectrum analysis	cTnI	5 pg/ml	[39]
New spectrum analysis	cTnI	10 pg/ml	[40]
Terahertz sensing	Troponin	0.5 μg/ml	[41]
Terahertz sensing	cTnI	0.5 pg/ml	This work

## Discussion

In this work, we present the exceptional capabilities of the designed metasensor system for trace detection both theoretically and experimentally. The integration of TPX-based split-ring resonators with functionalized AuNPs has significantly amplified the efficiency of molecular interactions between the analyte and the biosensor. The *Q*-factor and sensitivity of our *q*-BIC-based metasurface can be further improved by adjusting the asymmetric characteristics of open-ring resonators. Despite limitations in machining accuracy, the proposed metasurface achieves a sensitivity of up to 560 GHz/RIU. In the detection of cardiac troponin, we have surpassed expectations by achieving a remarkably low-concentration limit of 0.5 pg/ml, showing an order of magnitude lower than current methods. Our exceptional sensitivity metasurfaces hold promise for trace detection without requiring complex preparation, thereby advancing the development and application of THz metasensors in early disease diagnosis.

In short, THz waves have received increasing attention due to their unique advantages, including nonionizing biocompatibility and fingerprint spectral characteristics. In biomedical applications, THz spectroscopy covers molecular vibrational features, enabling identification and characterization of biomolecules and offering noninvasive, label-free identification and modulation of living cells. Recent research has shown that noninvasive THz targeted stimulation of the auditory cortex in mice enhances learning speed by 50% [32], attributed to the activation of ion channels in neurons [33]. This groundbreaking discovery reveals a novel approach to modulating protein behavior through THz waves. Thus, advancements in sophisticated THz detection tools are crucial for early diagnosis, monitoring cellular activities, and human healthcare.

## Materials and Methods

### Numerical simulations

The transmission spectra and near-field electric distributions of the micro-antenna arrays are computed using the frequency domain solver of the CST Studio suite software. The simulation process involves 6 main steps: establishing the physical model, defining materials, setting up the simulation region, configuring light sources, adding monitors, and executing simulations. The periodicity of the arrays is interpreted using periodic Floquet boundary conditions defined for the array unit cell. The open boundary condition is adopted in the *z*-direction. The electric field of the excitation THz wave is polarized along the *y*-axis. The substrate material is set as a lossless medium and the resonator is modeled as a PEC. The simulation mesh is set with a size smaller than 1/10 of the minimum working wavelength in order to obtain a high numerical accuracy.

### Metasensor microfabrication

Fabrication process: (a) Preparation of TPX substrate: The foundational substrate undergoes a process involving injection molding and precise engraving to attain a desired thickness of 2 mm. The effective refractive index of the polymer post-molding is approximately 1.46. (b) The intricate fabrication of the metasurface featuring open-ring resonators unfolds in the subsequent steps: (i) Manufacturing begins with the cleaning and drying of the 2-mm TPX substrate. (ii) Employing UV photolithography using a positive photoresist (RZJ-304, viscosity: 10 mPa⋅s) on the TPX, succeeded by the sequential deposition of a 10-nm titanium layer and a 100-nm gold layer via magnetron sputtering. Titanium acts as an adhesion promoter to augment bonding between the gold film and TPX. The exposure duration stands at a precise 26 s, exerting a critical influence on the dimensions and quality of the structure. (iii) Executing a lift-off process, culminating in the formation of the patterned resonator array.

### THz spectroscopy system analysis

The ultra-broadband spectroscopic data were obtained utilizing the THz TAS7500TS system purchased from Advantest Co., Ltd, Tokyo, Japan. This system primarily comprises fiber lasers and data acquisition modules. A synchronized control system emits 2 slightly different frequency femtosecond lasers, emitting and detecting THz waves through asynchronous sampling. The output power of the lasers exceeds 20 mW with a wavelength of 1,550 nm, a repetition rate of 50 MHz, and a pulse width of less than 50 fs. The system employs phase-modulated dual-laser measurement to capture time-domain signals containing essential details of the analyzed substances. It achieves a frequency accuracy of ±10 GHz and a minimum spectral resolution of 3.8 GHz, corresponding to time scanning ranges of 262 ps. The emitted THz signal in transverse magnetic polarization mode was illuminated on the metasensor. To ensure dependable sensing results and mitigate the impacts of the surrounding environment, an air dryer was employed to dry the experimental environment, ensuring that the relative humidity remained below 5%.

### Immunofunctionalization of AuNPs

In the experiments, we utilized several primary biological samples: cardiac troponin I and its antibodies were commercially prepared by Bioss (Beijing, China). The BCA protein assay kit was procured from ThermoFisher (Massachusetts, USA). The high-sensitivity covalent conjugation AuNPs assay kit was obtained from nanoComposix (San Diego, USA). Prior to conducting the experiments, the concentration of cardiac troponin I antibodies was verified using BCA Protein Assay Kits. The antibodies were purified through a desalting column and filter device. Carboxyl AuNPs (40 nm in diameter) were activated by vortexing and incubating with EDC/NHS for 30 min. After removing the supernatant, the complex AuNPs were resuspended in 1 ml of potassium phosphate (pH 7.4) reaction buffer. Antibodies were added to the particles and incubated for 1 h, followed by quenching with hydroxylamine for 10 min at room temperature. Each nanoparticle was washed by centrifugation at 3,800 relative centrifugal force for 10 min with reaction buffer, then resuspended in NCX conjugate diluent and stored at 4 °C. A series of preset concentration solutions (ranging from 1 mg/ml to 1 pg/ml) of cardiac troponin I and the coupled immunofunctionalized AuNPs were prepared. These solutions were vortexed and incubated at room temperature for 30 min before being applied to the metasurface for analysis.

## Data Availability

The data that support the findings of this study are available from the corresponding authors upon reasonable request.

## Supplementary Materials

Supplementary 1 Texts S1 to S4 Figs. S1 to S4
