# Supplementary material for: Ultrasensitive Terahertz Label-Free Metasensors Enabled by Quasi-Bound States in the Continuum
Source: Research (Wash D C). 2024 Sep 26;7:0483. doi: 10.34133/research.0483 (PMC11425342; doi:10.34133/research.0483)
Supplement: Supplementary 1 — Texts S1 to S4 Figs. S1 to S4 [file research.0483.f1.docx]

Supporting Information

**Ultrasensitive Terahertz Label-Free Metasensors Enabled by Quasi-Bound States in the Continuum**

Ride Wang, Lingyu Song, Hao Ruan, Quanlong Yang, Xiao Yang, Xiaobao Zhang, Rundong Jiang, Xiangmin Shi, and Alexander P. Shkurinov

**S1. The simulation calculation of the THz metasurface at *g*_2_ = 8 μm**

The sharp resonance dip at *f* = 1.94 THz in Figure S1a represents the quasi-BIC (*q*-BIC) in the reflection spectrum. Upon the multipolar decomposition of the structure at *g*_2_ = 8 μm in Figure S1b, the resonance mode is dominated by strong magnetic dipole (MD) mode, thereby reinforcing the analysis observed at *g*_2_ = 4 μm.

**
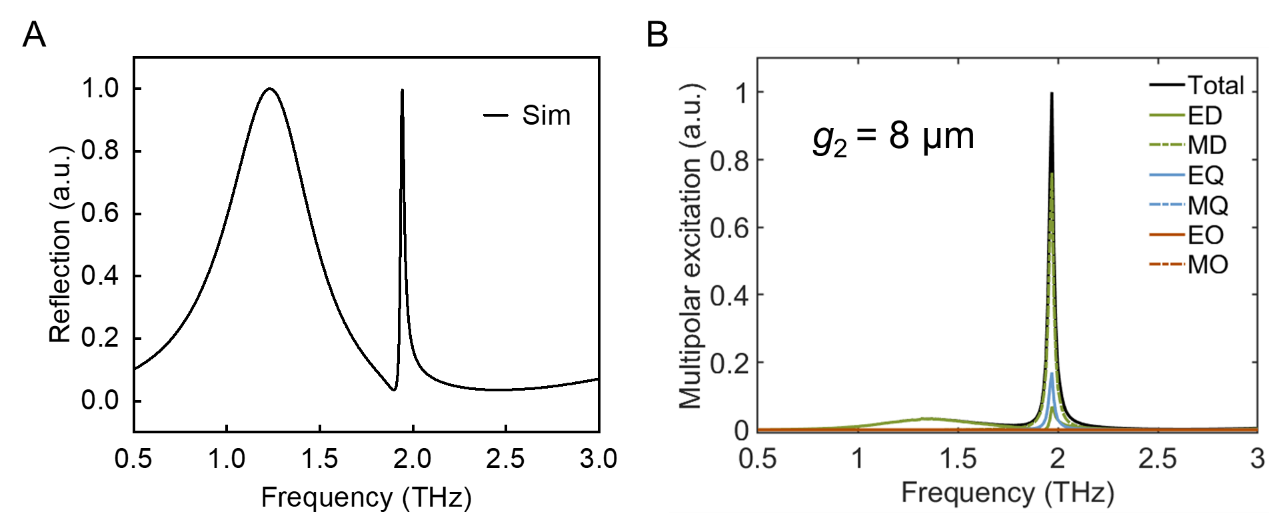
**

**Fig. S1.** The simulated reflection spectrum and multipolar decomposition results at *g*_2_ = 8 μm.

**S2.** **The simulation of charge density distribution**

In the case of ED resonance at *f* = 1.18 THz in Figure S2a, the enhancement of the electric field primarily concentrated within the connecting rod inside the resonator. Conversely, the local electric field of *q*-BIC resonance at *f* = 1.89 THz in Figure S2b is mainly concentrated in the resonator edge and the large gap region, implying a heightened potential for interaction with the analyte.

**
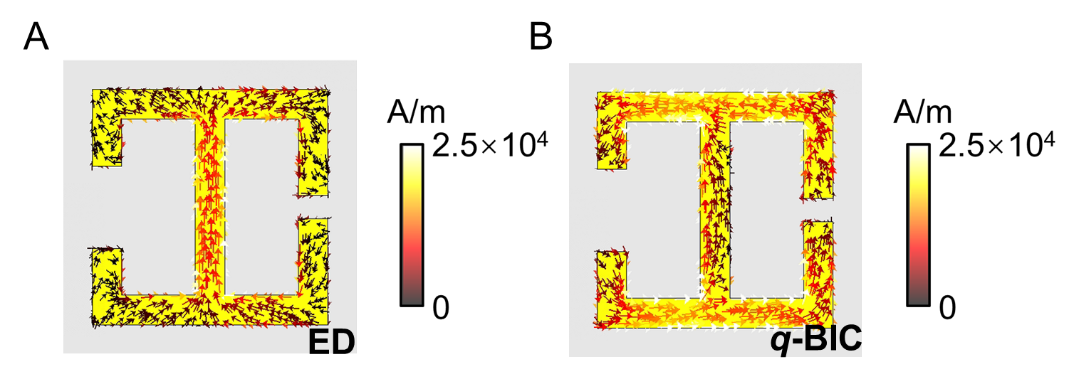
**

**Fig. S2.** The charge density distribution of ED resonance and *q*-BIC resonance at *g*_2_ = 4 μm.

**S3. Schematic illustration of the experimental setup**

**
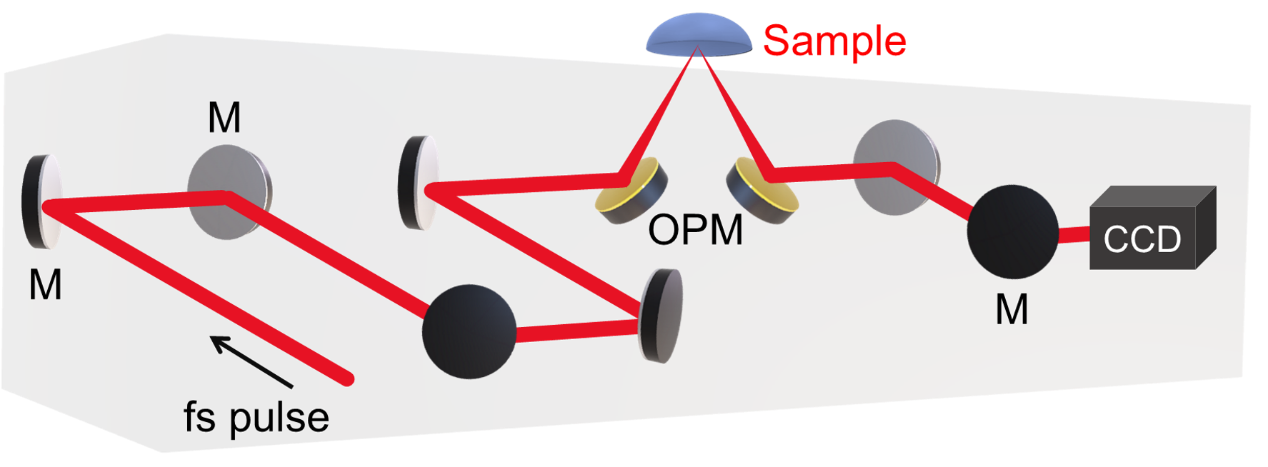
**

**Fig. S3.** The diagram of the optical path. M denotes mirrors; OPM represents off-axis parabolic mirrors; CCD signifies charge-coupled device.

**S4. Characterization of different concentrations of cardiac troponin I (cTnI)**

**
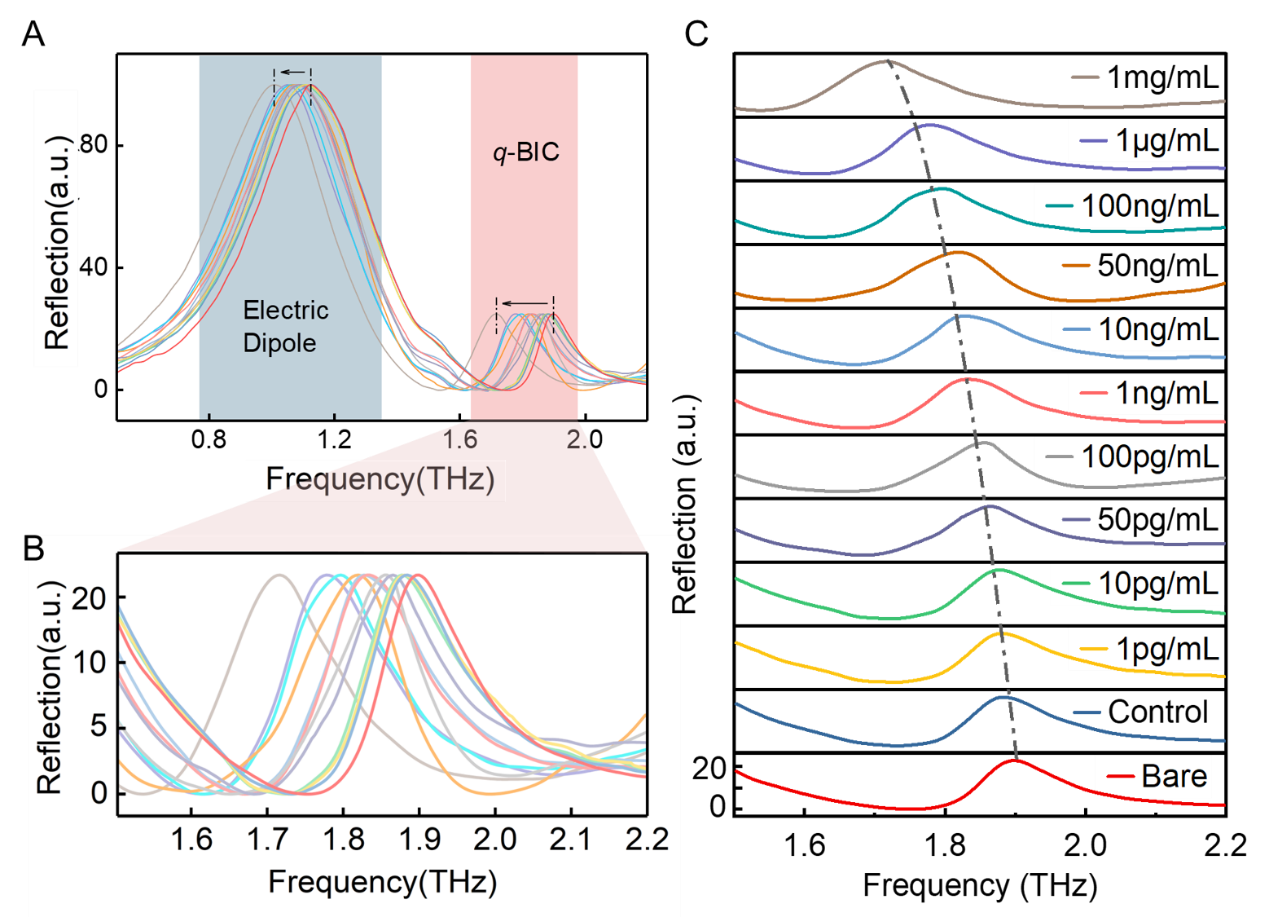
**

**Fig. S4.** The comprehensive reflection spectra of the *q*-BIC mode at various concentrations of the cTnI solution.
